# Modeling of energy consumption factors for an industrial cement vertical roller mill by SHAP-XGBoost: a "conscious lab" approach

**DOI:** 10.1038/s41598-022-11429-9

**Published:** 2022-05-09

**Authors:** Rasoul Fatahi, Hamid Nasiri, Ehsan Dadfar, Saeed Chehreh Chelgani

**Affiliations:** 1 School of Mining Engineering, College of Engineering, University of Tehran, Tehran, 16846-13114 Iran; 2 Department of Computer Engineering, Amirkabir University of Technology (Tehran Polytechnic), Tehran, Iran; 3 Production Department of Ilam Cement Plant, Ilam, Iran; 4 Minerals and Metallurgical Engineering, Department of Civil, Environmental and Natural Resources Engineering, Luleå University of Technology, SE-971 87 Luleå, Sweden

**Keywords:** Energy science and technology, Engineering

## Abstract

Cement production is one of the most energy-intensive manufacturing industries, and the milling circuit of cement plants consumes around 4% of a year's global electrical energy production. It is well understood that modeling and digitalizing industrial-scale processes would help control production circuits better, improve efficiency, enhance personal training systems, and decrease plants' energy consumption. This tactical approach could be integrated using conscious lab (CL) as an innovative concept in the internet age. Surprisingly, no CL has been reported for the milling circuit of a cement plant. A robust CL interconnect datasets originated from monitoring operational variables in the plants and translating them to human basis information using explainable artificial intelligence (EAI) models. By initiating a CL for an industrial cement vertical roller mill (VRM), this study conducted a novel strategy to explore relationships between VRM monitored operational variables and their representative energy consumption factors (output temperature and motor power). Using SHapley Additive exPlanations (SHAP) as one of the most recent EAI models accurately helped fill the lack of information about correlations within VRM variables. SHAP analyses highlighted that working pressure and input gas rate with positive relationships are the key factors influencing energy consumption. eXtreme Gradient Boosting (XGBoost) as a powerful predictive tool could accurately model energy representative factors by R-square ever 0.80 in the testing phase. Comparison assessments indicated that SHAP-XGBoost could provide higher accuracy for VRM-CL structure than conventional modeling tools (Pearson correlation, Random Forest, and Support vector regression.

## Introduction

As one of the most energy-intensive industries, cement plants consume around 100 kWh of electrical energy for each ton of their production. This can be counted yearly as over 6% of global energy consumption. More than 60% of this tremendous energy has been used in the comminution units (crushers and mills) to reduce the size of raw materials and clinker^1–3^. In the mid-1990s, the vertical roller mill (VRM) was introduced to the cement industry to reduce this energy usage. Besides lowering power consumption, VRMs may improve process capacity and simplify it since VRMs can simultaneously implement milling and drying processes. However, controlling VRM performances and understanding relationships within their operational variables need serious attention^4–7^.

In the cement plant, the conventional VRM controlling systems mainly rely on the field staff to manually adjust the few process parameters based on their experience. These adjustments generally lead to having an unstable system, increasing power consumption, and reducing plant productivity^8^. For filling these gaps and having a long-term stable operation, it would be essential to provide a complete picture of relationships within VRM elements. Understanding these correlations and their magnitude would help develop models for generating robust controlling systems. Generating such systems and assessing the VRM process operational parameters would help to optimize power consumption, improve maintenance, reduce environmental issues, and make the process sustainable.

A few investigations have been conducted to model VRM performance. Fernandes et al.^9^ used the back-propagation neural network (BPNN) to model size products of a raw VRM mill. They indicated RMSprop as an optimizer for modeling raw meal residual values would generate a lower error than the Adagrad and Adam optimizers. Their results showed that BPNN algorithms could accurately predict raw meal residue product quality in the cement industry. The population balance model for simulation of a VRM in a cement clinker grinding circuit was investigated by Fatahi and Barani^11^. They reported that the clinker particle spent a short time inside the VRM, and the mean residence time is about 67 s. The tanks-in series model compared to the Weller model was more proper to describe the residence time distributions in the VRM^11^. Extreme learning as an artificial intelligence (AI) method was used for modeling online measurement quality parameters of a raw material VRM. Results showed that the proposed model effectively achieved the online estimation of the key indicator parameters for the VRM process, laying the foundation for online parameter optimization^11^. However, no published study has been modeled and examined inter-correlations between VRM energy consumption indicative factors and plant operational parameters. Using “conscious lab (CL)” and constructing models based on operational data originated from industrial VRM could be an innovative way to tackle these gaps.

CL as a new model vision constructs based on datasets that have been generated by monitoring operational parameters within industrial plants^12–15^. CL is exploring relationships between these parameters by using AI systems and highlighting the effectiveness of each variable on the key process factors^16,17^. CL can be upgraded using explainable artificial intelligence (EAI) models as an innovative concept. EAI systems are emerging approaches of modeling that translate big complicated datasets into know-how information and relieve considerable transparency by converting complex relationships into human basis structures^18,19^. In other words, EAIs can change black box AI systems into white-box ones. CL constructed using EAI models and real processing datasets can be a powerful tool for ranking operational variables based on their importance, reducing time, cost, and possible laboratory and scale-up errors, and can be considered for training operating person based on the plant reality.

As a strategic approach, for the first time, this investigation is going to develop a CL based on over 3000 records monitored from a cement VRM circuit by using the most recent generated EAI models called "SHAP" (SHapley Additive exPlanations). Based on the game-theoretic approach, SHAP explores relationships within variables (linearly and nonlinearly), ranks them based on their importance, and marks their magnitude. SHAP illustrates these correlations for every record of variables and develops a complete explanation between the global average and the model output^20–23^. Besides SHAP, a sophisticated CL system would be required to predict the output variables accurately. Thus, XGBoost (eXtreme Gradient Boosting), a comprehensive predictive tool, has been employed to model motor power and outlet temperature as representative energy consumption factors of the VRM circuit. XGBoost is a flexible AI predictor tool with high performance and accuracy^22,24–27^. One of the main advantages of XGBoost over other typical machine learning methods is its more significant set of hyperparameters, which makes it capable of being better tuned. For comparison purposes, random forest^28–31^ and support vector regression^32–35^ as conventional AI methods have also been used to assess the SHAP-XGBoost ability to develop CL of VRM.

## Materials and methods

### Database

**Figure 1:**
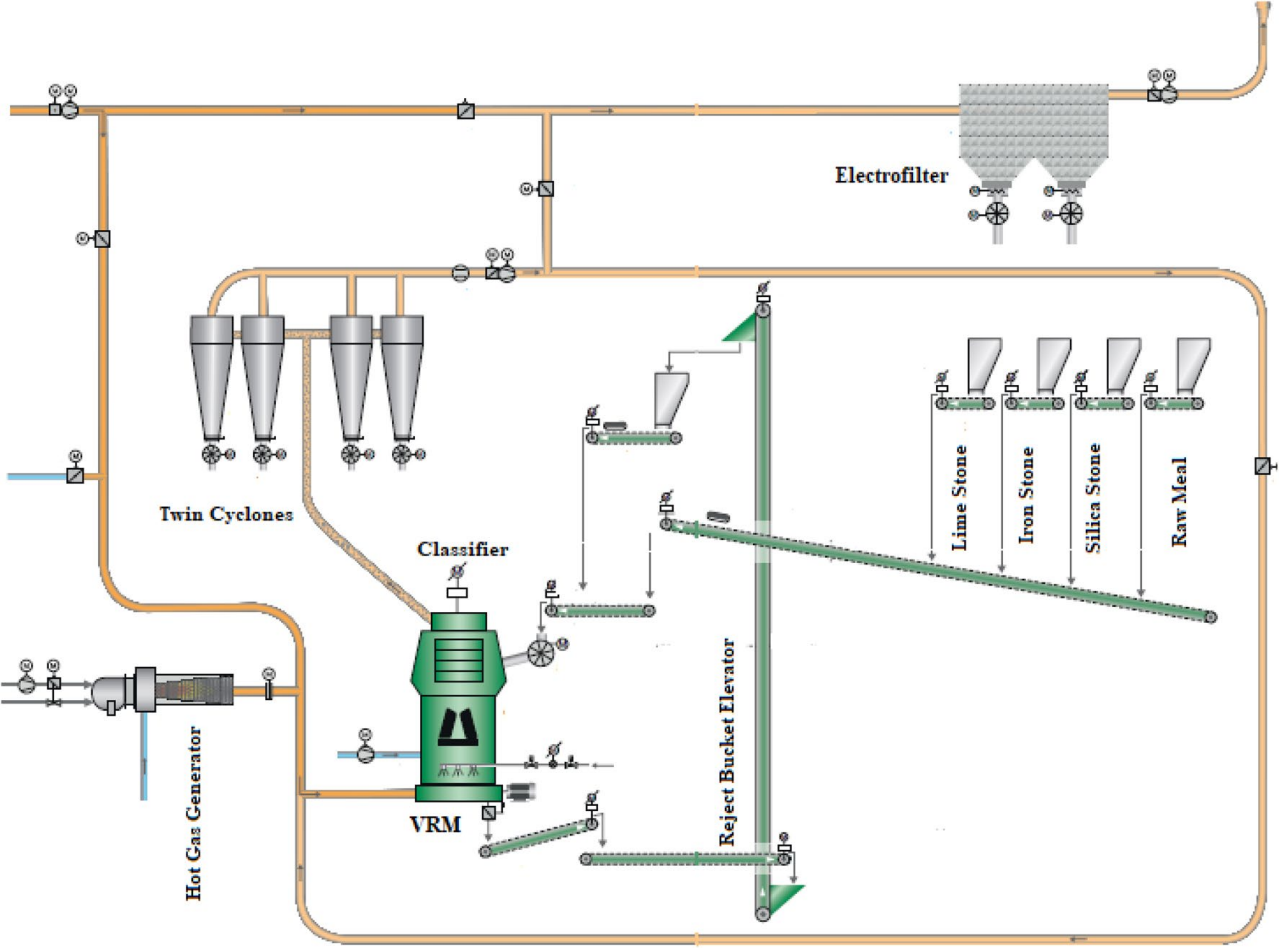
Schematic of raw vertical roller mill circuit in the Ilam cement plant.

The provided data were collected from a cement plant (Fig. 1) located in Ilam, west of Iran. The plant has two cement production lines which in total produces 5300 t/day cement. The raw materials (lime, silica, and iron ore) enter the circuit through two apron feeders. The raw materials are crushed in a hammer crusher to D_95_ 80 mm. The raw materials were mixed in a certain proportion and fed into a vertical roller mill (LOESCHE mill). The raw vertical roller mill has four rollers, 3000 KW main drive, 4.8 m table diameter, 2.16 m roller diameter with 330 t/h capacity (made by LOESCHE Company from Germany). The table mill's rotation speeds are mainly constant, and there is approximately a fixed one-year period of changing liners of the mill body and hardfacing operations of wear rollers. For constructing a CL dedicated to the VRM circuit and predicting motor power and outlet temperature (as indicative energy factors), a dataset was collected from one of the vertical roller raw mill circuits (line 2) in the Ilam cement plant. The critical operating parameters gathered during the standard operation are summarized in Table 1. Variables were monitored hourly and were taken into account. In general, over 3000 records were prepared and used for the modeling.

**Table 1:** Monitoring variables in the Ilam cement plant (STD: Standard deviation).

Description of variables	Variables	Min	Max	Mean	STD
The load of mill feed	Feed rate (ton/h)	250	307	297.16	7.11
The applied pressure for grinding by roller	Working pressure (bar)	68	80	72.76	1.92
The required hot gas for drying and transportation of raw material	Input gas flow (m^3^/h)	60,000	890,000	600,559	90,434
The speed of classifiers rotor	Classifier speed (rpm)	48	59	52.47	1.03
The vibration of mill body due to operational parameters	Mill body vibrating (mm/s)	2.30	36.90	4.16	1.13
The temperature of the mill inlet	Input temperature (°C)	21	2132	204.50	61.26
The differential pressure between inlet and outlet of mill	ΔP (mbar)	9	97	85.37	3.45
The pressure of the mill inlet	Input pressure (mbar)	− 6	4	− 2.27	0.88
The temperature of the mill outlet	Output temperature (°C)	7	98	66.99	9.82
The power drawing of the main motor	Motor power (kW)	138	2370	1845.76	397.32

In the LOESCHE mill, the rollers are hydraulically pressed against a disc table, and the feed would be crushed and pulverized between the rollers and the disc table. The motor power running all four rollers was calculated based on Eq. (1). In other words, the rollers are hydraulically pressed by working pressure against a table, and the feed is ground between the rollers and the table^36–38^. Therefore, working pressure would affect the size distribution of products. The main motor power is related to the rollers' applied pressure (working pressure) and the feed rate of raw materials on the grinding table. The hot gas was produced by kiln and preheater^7^. For drying, ground materials are transported to the separator by hot gas that is introduced into the mill. Thus, the difference between the input and output pressures of the mill (ΔP) would be essential. Dried material will be transferred for size classification^37^. Unground material would stay over the classifier, and they have to be kept inside the mill to meet the desired size. One of the critical factors through the process is controlling the mill body vibrating as a result of the working pressure of the rollers on the crushing table^39^. After the grinding, drying, transportation, and separation process inside the mill, the product is transferred as cement kiln feed to a storage silo.

1 $$P=\sqrt{3 }IVCos\varphi$$

where Cos $\varphi$ (Cos $\varphi$ is 0.88 for the Ilam cement production) is the power factor, I is the current, V is the voltage, and P is the power.

### Modeling

**Figure 2:**
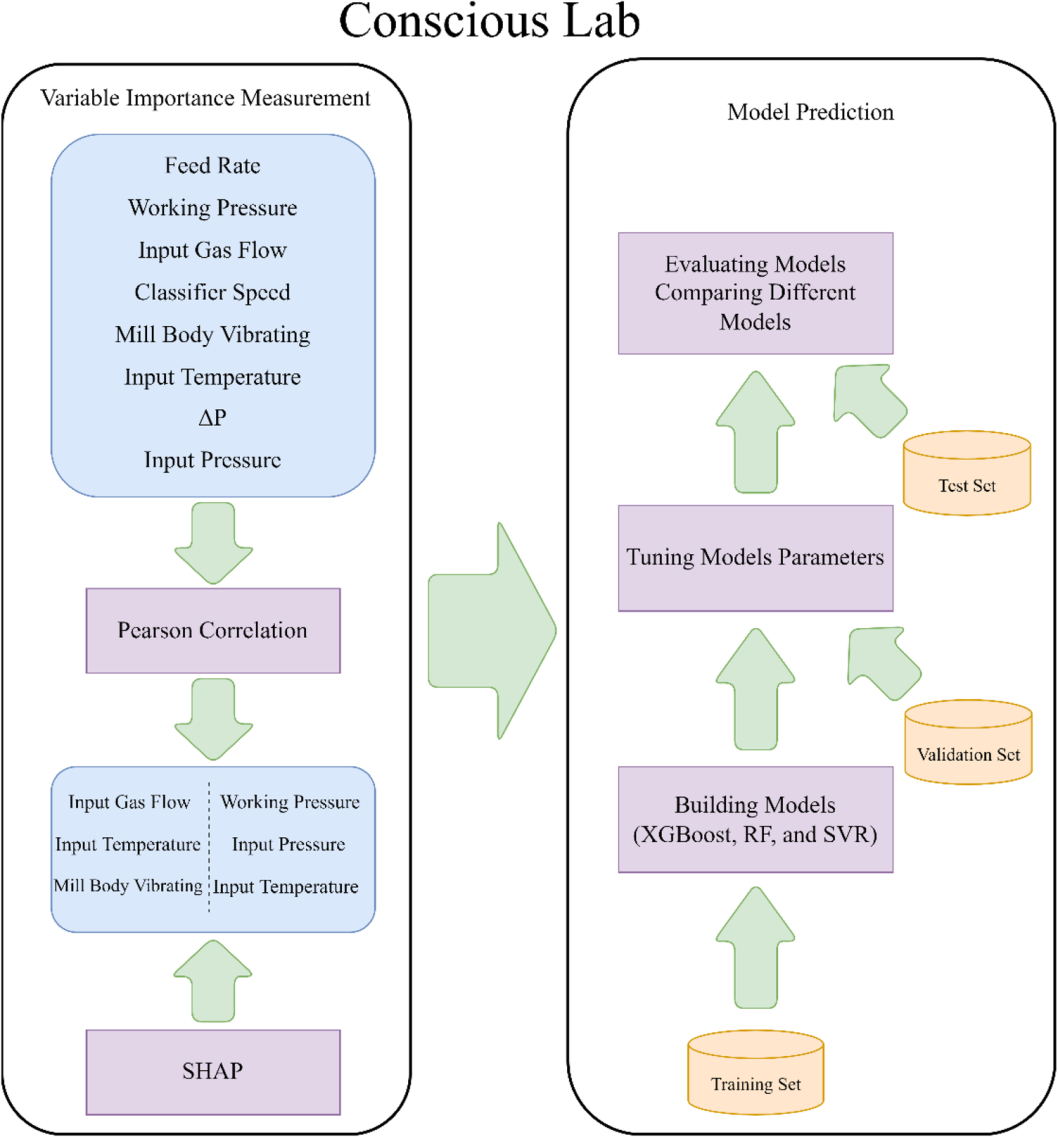
Constructing a conscious lab for a vertical roller mill.

After removing the missing data, the provided dataset was processed by different AI models. SHAP and Pearson correlation were initially considered to assess the relationship between variables. After that, the dataset was randomly split into three sections: training (70%), validation (15%), and testing (15%). Similar dataset sections were considered for constructing all the proposed models for comparison purposes. The procedure was based on the following diagram (Fig. 2).

#### SHapley Additive exPlanations (SHAP)

SHAP stands for "SHapley Additive exPlanations", a machine learning (ML) approach to explain models predictions and provide interpretability of an ML model. First presented by Lloyd Shapley, it uses Shapley values to interpret the model's output^16,23,40,41^. The Shapley value of a feature is equal to the difference between the average prediction value of samples with and without this feature^42^. It measures the feature's importance in the model^43,44^. Shapley value ${\phi }_{i}$ for the model $f$ can be computed as follows:

2 $${\phi }_{i}\left(f,\mathbf{x}\right)=\sum_{S\subset M\backslash i}\frac{\left|S\right|!\left(\left|M\right|-\left|S\right|-1\right)!}{\left|M\right|!}\left[f\left(S\cup \left\{i\right\}\right)-f\left(S\right)\right]$$

where $M$ represents the set of all input variables, $S$ denotes a subset of $M$ with the $i$ th feature excluded from $M$, and $f\left(S\cup \left\{i\right\}\right)-f\left(S\right)$ is the marginal feature contribution of the $i$ th variable^45–48^.

#### Extreme Gradient Boosting (XGBoost)

Extreme Gradient Boosting (XGBoost), proposed by Chen and Guestrin^49^, is an efficient and scalable ensemble algorithm based on gradient boosted trees^16,50^. XGBoost has been used in a wide range of engineering fields, resulting in outstanding performance due to the advantages of parallel tree boosting and using various regularization techniques^13,51,52^. XGBoost is a stable algorithm with low bias and variance, handling outliers^24,53^. It adds a regularization term to the objective function as follows:

3 $$Obj\left(\theta \right)=L\left(\theta \right)+\Omega (\theta )$$

where $L\left(\cdot \right)$ is a convex loss function and $\Omega \left(\cdot \right)$ is a regularization function used to avoid overfitting by controlling the model's complexity^54^. $\Omega (\theta )$ is calculated as follows:

4 $$\Omega \left(\theta \right)=\gamma T+\frac{1}{2}\lambda {\Vert w\Vert }^{2}$$

where $T$ denotes the number of leaf nodes, and $w$ is the weight of each leaf. $\gamma$ and $\lambda$ are regularization parameters that determine the relative weight of each penalty term^24,55–57^.

#### Random forest

Random forest (RF) is an ensemble learning technique that combines the bagged integrated learning theory^58^ with the random subspace approach^59,60^. RF is a nonparametric method, robust to outliers, and can handle missing values in data^16,61,62^. RF is a collection of decision trees that are grown independently. The predictions of these trees are aggregated by averaging to generate the final output. This ensures that the overall variance is reduced^24,56,63^. Mathematically speaking, RF generates an ensemble of $N$ decision trees. Using these trees, the final output of an input feature vector $\mathbf{x}$ is computed as follows:

5 $$\widehat{T}\left(\mathbf{x}\right)=\frac{1}{N}\sum_{n=1}^{N}{\widehat{T}}_{n}\left(\mathbf{x}\right)$$

where ${\widehat{T}}_{n}\left(\mathbf{x}\right)$ is the result of the $n$th tree’s estimation^13,64–66^.

#### Support vector regression

Support vector regression (SVR) is a nonparametric supervised machine learning approach proposed by Drucker^67^. Vapnik's support vector concept was the inspiration for Drucker to develop SVR^67^. An important feature of SVR is its powerful capability for nonlinear predictions^68^, which results from the nonlinear transformation it uses. SVR maps observations into a higher-dimensional feature space via nonlinear transformation and then solves the problem^24,69,70^. Given a training dataset with $n$ samples $T=\{\left({\mathbf{x}}_{1},{y}_{1}\right),\left({\mathbf{x}}_{2},{y}_{2}\right), ..., ({\mathbf{x}}_{n},{y}_{n})\}$, ${\mathbf{x}}_{i} \epsilon {\mathbb{R}}^{d}$, ${y}_{i} \epsilon {\mathbb{R}}$, the following linear function can formulate non-linear relation between input and output:

6 $$f\left(\mathbf{x}\right)={\mathbf{w}}^{T}\phi \left(\mathbf{x}\right)+b$$

where $f\left(\mathbf{x}\right)$ denotes the estimated output and $\phi \left(\mathbf{x}\right)$ is a mapping function. $\mathbf{w}$ and $b$ (i.e., bias) are two parameters that can be determined by optimizing the following objective function:

7 $$\phi \left(\mathbf{w},{\varvec{\xi}}\right)=\frac{1}{2}{\Vert \mathbf{w}\Vert }^{2}+C\sum_{i=1}^{n}({\xi }_{i}^{-}+{\xi }_{i}^{+})$$

where $C$ is the penalty parameter or regularization constant, ${\xi }_{i}^{-}$ and ${\xi }_{i}^{+}$ denote slack variables that represent the upper and lower constraints on the output variable^13,24,71–73^.

### Evaluation

Coefficient of determination (R^2^), Root mean square error (RMSE), and the differences between actual and predicted values in different stages of modeling (training, validating, and testing) were used to assess the model's accuracy.

8 $${R}^{2}=1-\frac{S{S}_{res}}{S{S}_{tot}}$$

where $S{S}_{res}$ denotes the sum of squares of residuals and $S{S}_{tot}$ is the total sum of squares that can be computed as follows:

9 $$S{S}_{tot}=\sum_{i}{\left({y}_{i}-\overline{y }\right)}^{2}$$

where ${y}_{i}$ and $\overline{y }$ represent the observed data and mean of the observed data, respectively. RMSE can be calculated as follows:

10 $$RMSE= \sqrt{\frac{{\sum }_{i=1}^{n}{\left({\widehat{y}}_{i}-{y}_{i}\right)}^{2}}{n}}$$

where ${\widehat{y}}_{i}$ and ${y}_{i}$ denote the predicted and observed values, respectively, and $n$ represents the number of samples. Moreover, to assess whether the performance of the XGBoost is statistically significant, a two-tailed Welch’s t-test with a significance level $\alpha =0.05$ was applied for RMSE and R^2^ between the XGBoost and other methods, and the obtained *p*-value was reported. Welch’s t-test is a nonparametric univariate statistical test used to test the hypothesis that two samples have equal means^74^.

## Results and discussions

### Relationship assessments

**Figure 3:**
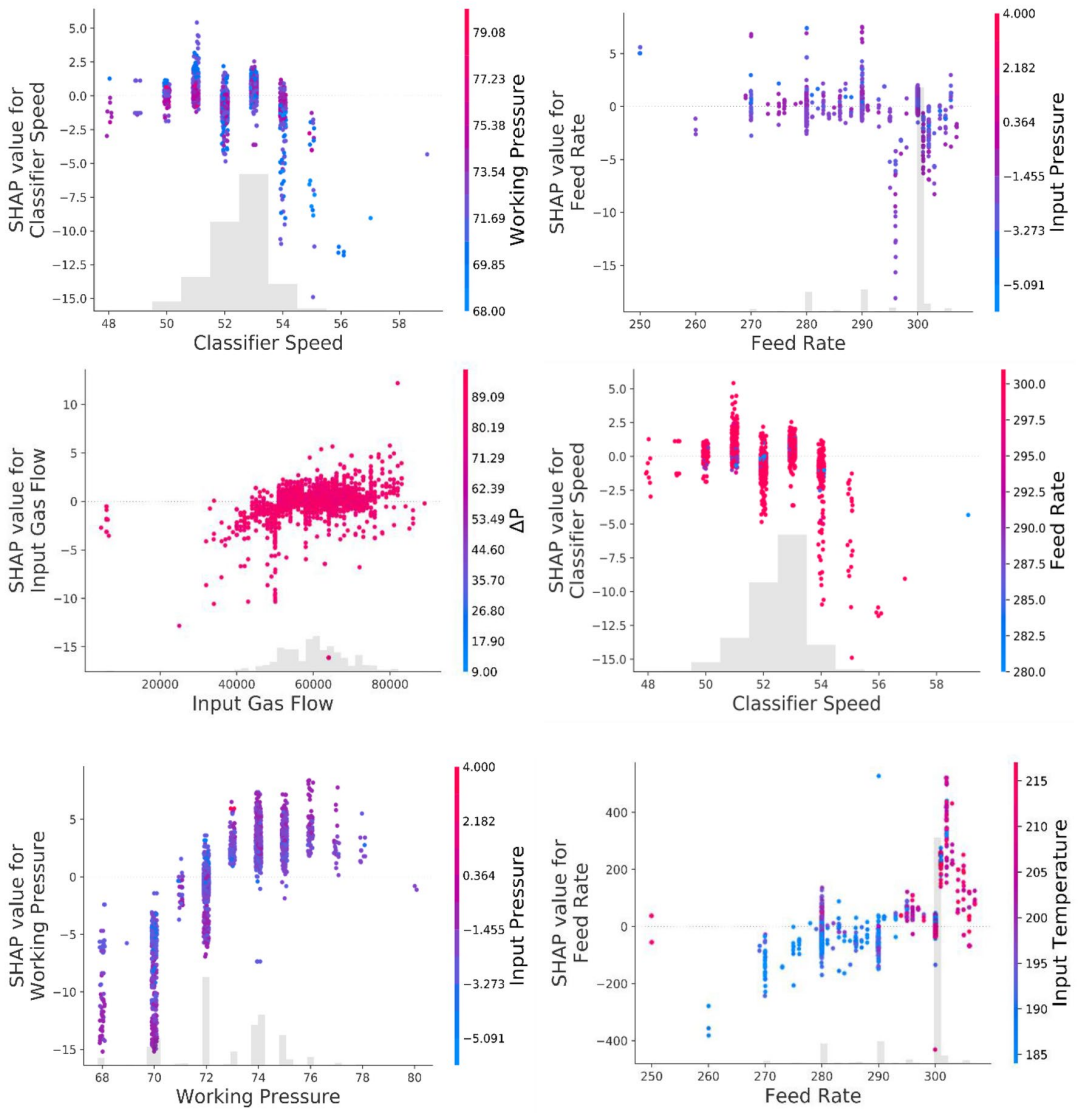
SHAP assessed the complexity of inter-correlations between VRM operational variables.

**Figure 4:**
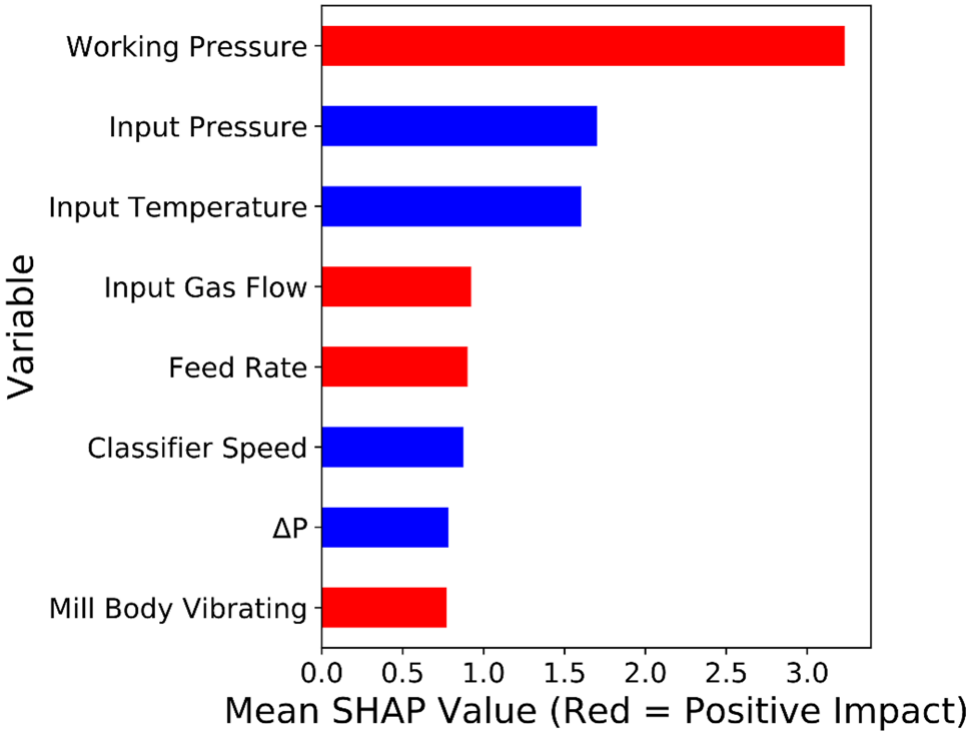
Ranking SHAP values and magnitude of relationships between VRM monitoring variables and output temperature.

**Figure 5:**
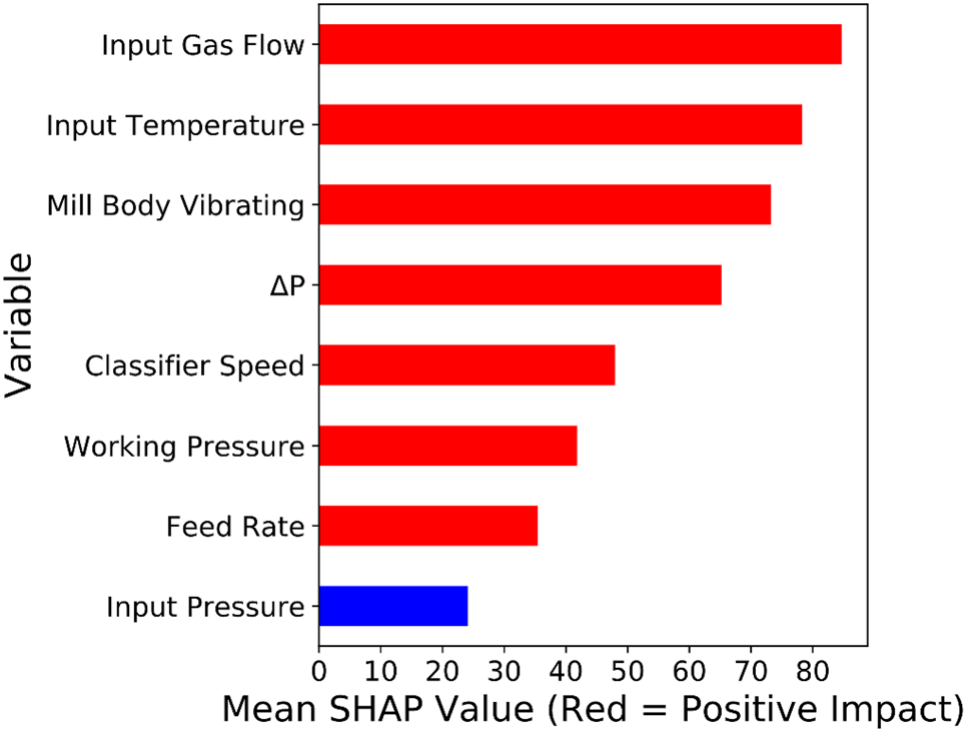
Ranking SHAP values and magnitude of relationships between VRM monitoring variables and motor power.

Exploring correlations and ranking variables based on their effectiveness on key parameters would help operate heavy machines such as VRMs accurately, make the process sustainable and reduce energy consumption. For drawing insights about relationships with the VRM variables, Pearson correlation (as a typical correlation assessment method) and SHAP assessment were conducted through the entire recorded data from the plant. SHAP (Fig. 3) showed the complexity of relationships between VRM operational variables. Ranking variables (Figs. 4, 5) based on their importance (SHAP values) illustrated that working pressure and input gas flow had the highest effectiveness on output temperature and motor power, respectively. Their correlations were positive. Working pressure (grinding pressure) could be considered the most effective variable through the VRM size reduction process. Increasing working pressure enhances the energy applied to the material, and more fines are offered to the classifier, leaving the circuit faster^37^. Altun et al.^6^ indicated a strong correlation coefficient between $\frac{Working pressure}{Classifier rotor speed}$ and product rate. In other words, increasing the work pressure would enhance energy consumption^6,7,37,75^.

**Figure 6:**
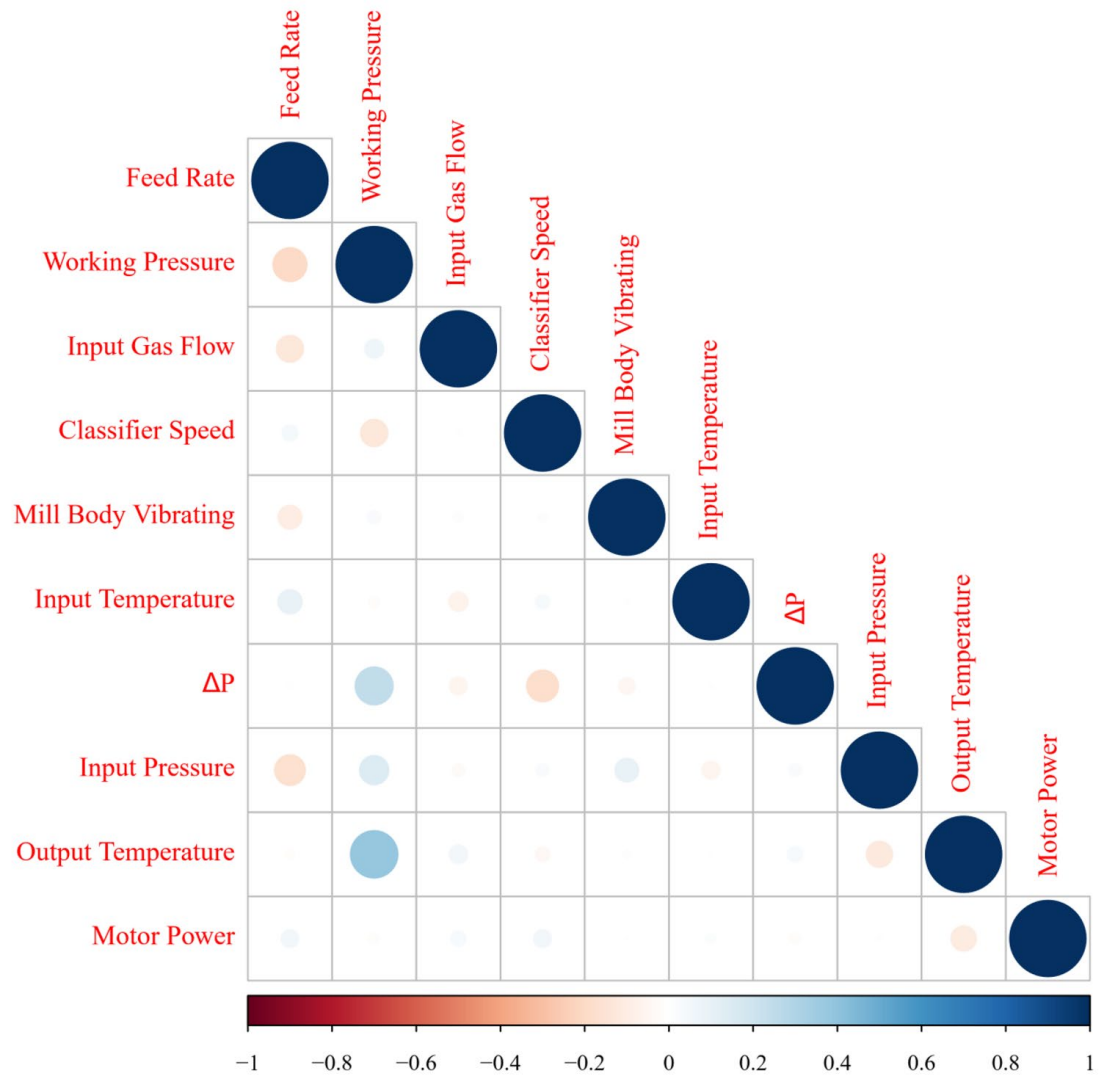
Pearson correlation assessments between VRM monitoring variables.

There is a good agreement between SHAP and Pearson correlation outcomes (Fig. 6); however, SHAP could model relationships much more accurately. It was well documented that Pearson correlation can only examine one by one linear relationship and show their magnitude. While, SHAP would develop a multi-linear-nonlinear interaction assessment among the recorded variables, rank them based on their importance, and highlight the magnitude of the multivariable relationships. For example, while linear relationship examination by Pearson correlation showed no significant interactions between input pressure or temperature and VRM indicative energy consumption factors, SHAP placed it within the most influential variables. Obviously, input temperature would affect the output temperature of products. Moreover, input pressure could commendably affect the process energy consumption since too low negative inlet pressure influences the steady gas flow within the system and disturbs the grinding procedure^6,37,75^. Pearson correlation showed a positive relationship between motor power, while multivariable assessment by SHAP illustrated a negative correlation. VRMs are very prone to vibration if their operational variables marginally are varied. It was reported that slight vibration could enhance particle transportation and improve energy consumption^7^. Apart from linear assessment (Fig. 5), gas flow also influences energy consumption factors, as SHAP illustrated (Fig. 4). Gas flow through the mill helps ensure constant lift for the internal circulating material and keeps separator performance constant to ensure a consistent product size distribution^6,76^.

**Figure 7:**
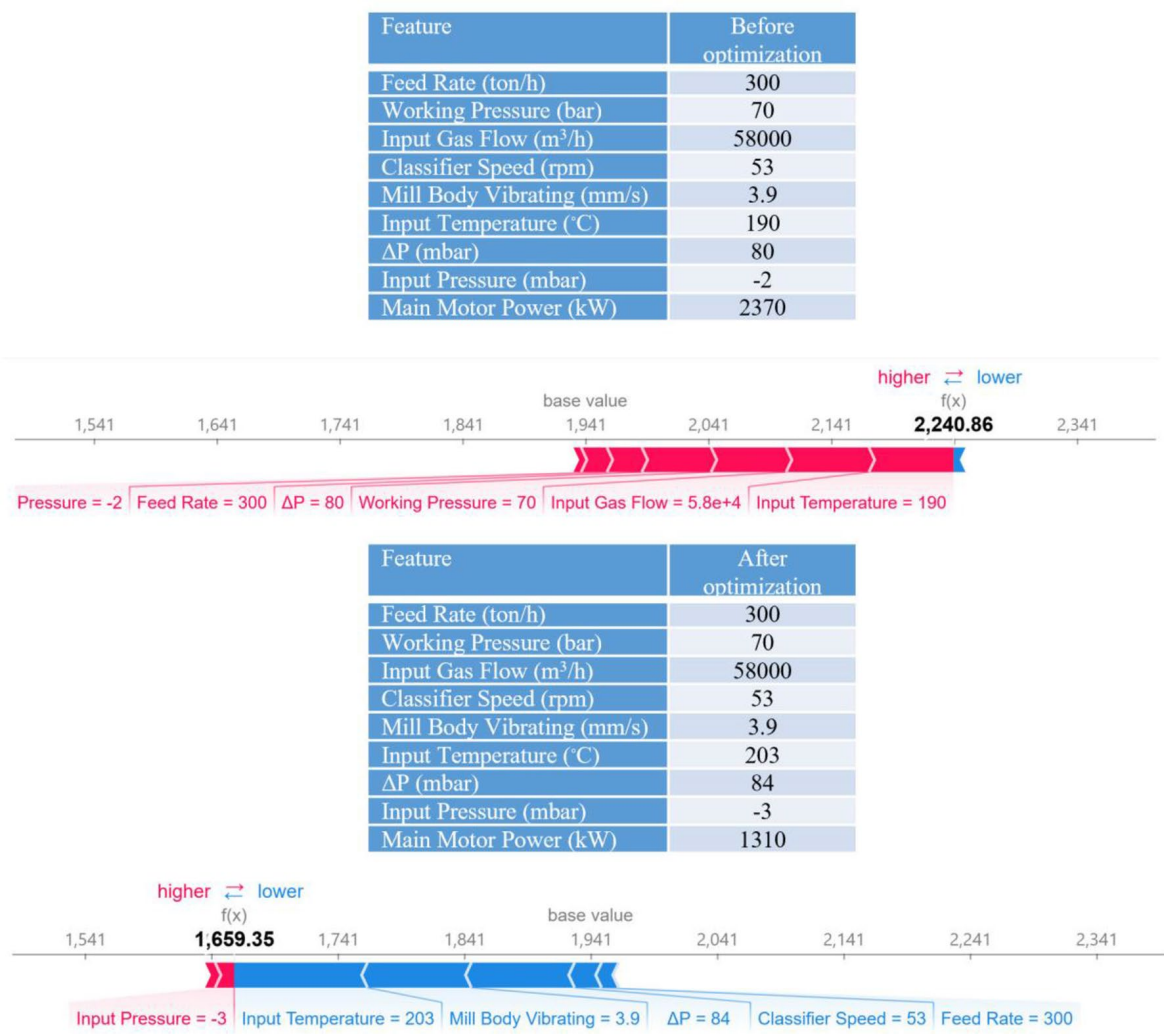
Possible optimization for the motor power consumption based on the SHAP results.

It was reported that only the mill input material feed rate has a decisive influence on the mill differential pressure (ΔP) while gas flow rate, grinding pressure, and classifier speed are maintained at the similar condition according to the pre-adjustments during operation unless the characteristics of the raw material such as the grind ability of the material have been changed^6,76^. However, SHAP results showed by increasing the ΔP, the power consumption was increased (Fig. 4). This correlation can be explained by the fact that variations in the ΔP when the grinding pressure and the hot air circulation are constant directly reflect the amount of material inside the mill. In other words, when the ΔP decreases, the amount of input material is less than the discharge material, causing the material bed to be thinner. Thus, as the ΔP increases, the material bed becomes thicker. VRM vibrates when the material bed is too thin or thick and trips or stops when the vibration limit is exceeded. For these reasons, the total feed amount must be adjusted so that the ΔP is within the correct range^6,76^. Based on these facts, SHAP analyses indicated (Fig. 7) that keeping the most effective parameter constant and changing other variables for the same size production makes it possible to reduce energy consumption. These results demonstrated that CL can model motor power and output temperature.

### Predictive models

**Figure 8:**
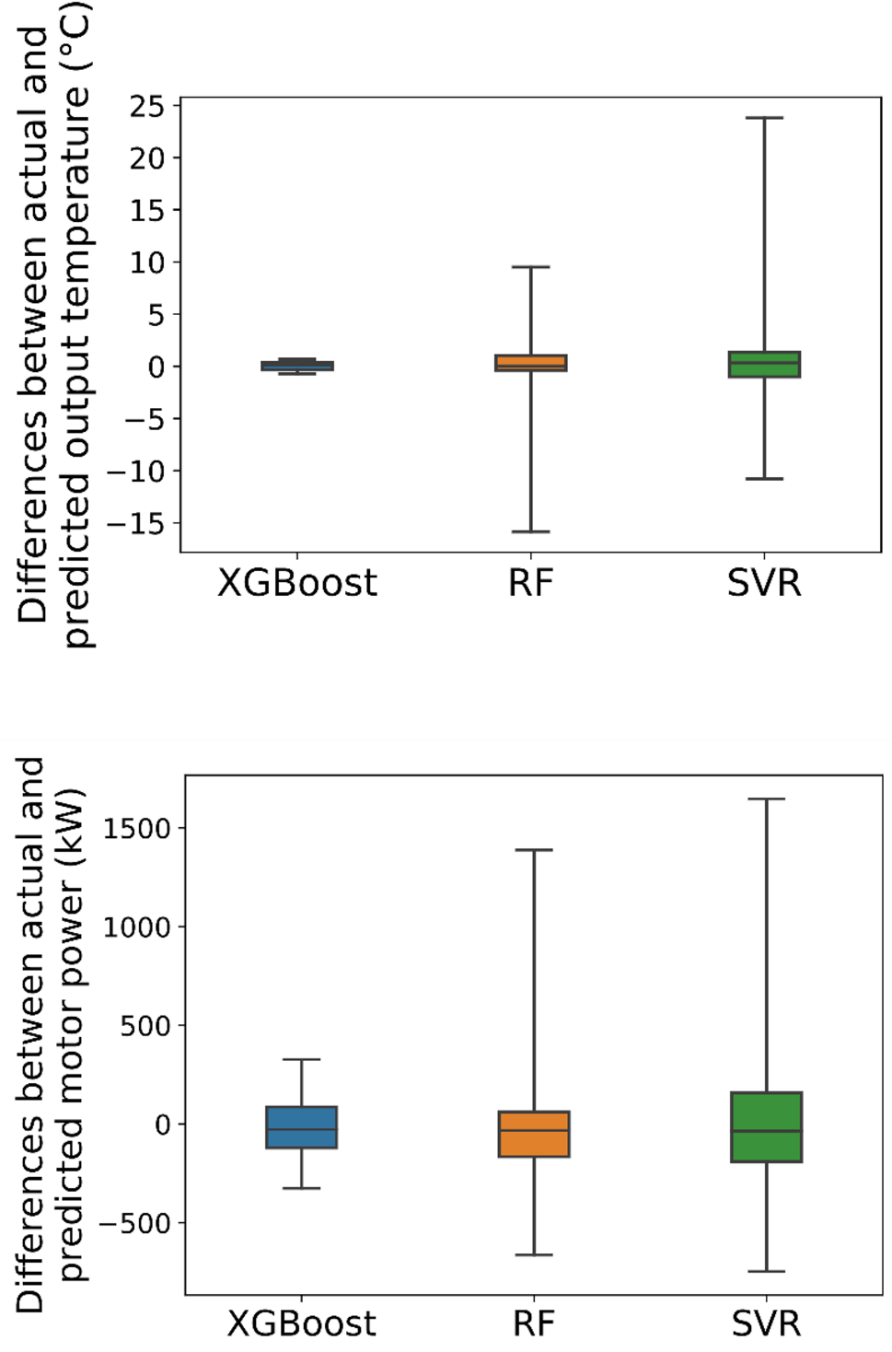
Differences between actual and predicted energy consumption indicative variables generated by various models in the testing phase.

For constructing predictive AI models (XGBoost, RF, and SVR), from the entire provided dataset, 70% of records were randomly used for the training step, 15% for the validation and the rest were considered for the testing step. Many XGBoost features were explored and adjusted during the training step for finding the most accurate models and tuning process (Table 2). The XGBoost validation and testing stage outcomes (Table 3) demonstrated that the generated model could quite accurately predict the energy consumption indicative essential factors based on the plant monitored variables. A comparison between various models' outcomes (Table 3) highlighted that the XGBoost model resulted in higher accuracy than these two conventional AI models for the prediction (Fig. 8). A two-tailed Welch’s t-test with a significance level $\alpha =0.05$ was applied for R^2^ and RMSE between the XGBoost and other methods, and the obtained *p*-value was reported in Table 3. As can be seen, in all comparisons, the null hypothesis is rejected based on the statistical tests with a 95% confidence level, and the results are considered statistically significant.

**Table 2:** The XGBoost parameter settings for predicting VRM indicative energy parameters.

Parameter	Value (output temperature)	Value (motor power)
Base learner	Gradient boosted tree	Gradient boosted tree
Tree construction algorithm	Exact greedy	Exact greedy
Learning objective	Regression with squared loss	Regression with squared loss
Learning rate ($\eta$)	0.260	0.225
Lagrange multiplier ($\gamma$)	6.53	1
Number of gradients boosted trees	60	81
Maximum depth of trees	16	7
The minimum sum of instance weight (Hessian) needed in a child	1	1
L2 regularization term on weights	1	1
The initial prediction score of all instances (global bias)	0.5	0.5
Subsample ratio of the training instances	1	1
Maximum delta step, we allow each leaf output to be	0 (there is no constraint)	0 (there is no constraint)

**Table 3:** Outcomes of various models in the validation and testing stages.

Method	Output temperature					
	R^2^			RMSE		
	Validation	Test	*p*-value	Validation	Test	*p*-value
**Random forest**						
Avg	0.88	0.83	3.69E − 09	2.42	2.56	6.58E − 14
std	(0.02)	(0.07)		(0.19)	(0.50)	
Support vector regression	0.49	0.39	1.82E − 168	4.88	4.94	3.78E − 185
XGBoost	0.99	0.99	–	0.36	0.41	–

	Motor power					
**Random forest**						
Avg	0.71	0.62	1.53E − 25	186.11	217.65	3.30E − 25
std	(0.01)	(0.01)		(2.54)	(3.59)	
Support vector regression	0.17	0.19	1.33E − 168	313.47	313.22	1.95E − 214
XGBoost	0.80	0.80	–	151.66	155.60	–

By comparing different machine learning methods used in this research, it is crucial noting that RF and XGBoost are both ensemble techniques, whereas SVR is not. XGBoost is a boosting method that builds on weak learners to train the next learner to enhance the already trained ensemble. RF is a bagging method that uses a random subset of features to train each weak learner independently. XGBoost and SVR have a low computational cost, but RF does not. SVR takes advantage of the kernel trick, and XGBoost uses parallel processing to reduce the computational cost. All three methods are getting little impact from outliers. XGBoost and RF are performed well with missing data in the dataset, but SVR does not. SVR has low bias and high variance in terms of bias and variance, while XGBoost and RF have low bias and variance^24,53,77–80^. These outcomes illustrated that SHAP-XGBoost could effectively construct a CL for a VRM circuit as an impressive EAI structure. Moreover, these results showed that using EAI can highlight the reality of relationships between operating variables on the industrial scale. Therefore, besides controlling the system regarding the process variables, it would be possible to predict the performance of existing machines based on the new feed materials, reduce penalties and keep the circuit sustainable. The robust capability of such a system depicted the potential of industrial digitalization for understanding, predicting, and maintaining various powder technology processes and controlling their energy consumption.

## Conclusion

Understanding relationships among operational variables can effectively help to improve control systems and reduce energy consumption in the cement plant as one of the most intensive energy consumer industries. Digitalization and constructing a conscious lab for exploring correlations between operational variables of a vertical roller mill and its indicative energy factors would potentially enhance its maintenance and efficiency. SHAP-XGBoost, as one of the most recently developed explainable artificial intelligence systems, would be a novel approach for developing a conscious lab and converting industrial datasets to understandable human basis pictures. SHAP-XGBoost could accurately depict correlations among operational parameters of an industrial vertical roller mill. SHAP assessment indicated that working pressure and input gas flow had the highest effectiveness (positive correlations) on output temperature and motor power, respectively. Pearson correlation and SHAP could highlight a negative inter-correlation between classifier speed and working pressure. Moreover, results showed that increasing the input gas flow would decrease the input temperature. XGBoost has accurately estimated the vertical roller mill's output temperature and motor power based on the plant monitoring variables (R-square over 0.99, and 0.80 for the output temperature and motor power, respectively). In the validation and testing stages, a comparison between results of SHAP-XGBoost and the other examined conventional models (Pearson correlation, random forest, and support vector regression) indicated that SHAP-XGBoost as a powerful method could be applied for generating conscious labs which dedicated to the energy sector factors within powder production technologies.

## Data Availability

The dataset used to support the findings of this study is available from the corresponding author upon request.
